# Efficiency of blood utilization in elective surgical patients

**DOI:** 10.1186/s12913-019-4584-1

**Published:** 2019-11-06

**Authors:** Kibruyisfaw Zewdie, Abraham Genetu, Yeabsera Mekonnen, Tewodros Worku, Abat Sahlu, Dereje Gulilalt

**Affiliations:** 1 Department of Surgery, Neurosurgery Unit, Tikur Anbessa Specialized Hospital, Collage of Health Science, Addis Ababa University, P.O. box 9086, Addis Ababa, Ethiopia; 2 Department of Surgery, Cardiothoracic Unit, Tikur Anbessa Specialized Hospital, Collage of Health Science, Addis Ababa University, P.O. box 9086, Addis Ababa, Ethiopia

**Keywords:** Efficiency of blood transfusion, Transfusion index, Transfusion probability, Tikur Anbessa hospital Ethiopia

## Abstract

**Background:**

Requesting blood prior to a surgical procedure for perioperative transfusion is a common practice in surgical patients. More unit of blood is requested than used by anticipating the patient will be transfused to provide a safety margin in an event of unexpected haemorrhage. Over requesting with minimal utilization results in significant wastage of blood, reagents and human resource. This study was conducted to assess blood utilization practice of the largest tertiary hospital in Ethiopia.

**Methods:**

A cross-sectional prospective study method was used. Data was collected using a Proforma questionnaire by perusal of each individual patient’s records from December 1, 2017 to February 28, 2018.patient age, sex, department requesting the blood, level of operating surgeon, hemodynamic status, number of unit requested, number of unit crossed matched and number of unit transfused were collected. Efficiency of blood utilization was calculated with three indices: Crossmatch to transfusion ratio, transfusion probability, and transfusion index indices.

**Results:**

Blood was requested for 406 patients and a total of 898 units were crossmatched for this patients. Overall Crossmatch to transfusion ration, transfusion probability and transfusion index were 7.6, 15.3% and 0.29 respectively. Results showed insignificant blood usage. Among different departments and units, better blood utilization was seen in neurosurgical unit with C/T ratio, TP and TI of 4.9, 24.4 and 0.6% respectively, while worst indices were from obstetrics unit with C/T ratio, TP and TI of 31.0, 6.5% and 0.06.

**Conclusion:**

Using all the three parameters for evaluation of efficiency of blood utilization, the practice in our hospital shows ineffective blood utilization in elective surgical procedure. Blood requesting physician should order the minimum blood anticipated to be used as much as possible.

## Background

The written history of blood transfusion dates back to 1666 when Richard Lower conducted experiments involving transfusion of blood from one animal to another [1]. However, it was James Blundell, who was an Obstetrician in London, who is credited with being the first person to transfuse blood from one human to another successfully [2]. Later, the discovery of ABO blood groups by Landsteiner (1901) as well as subsequent other antigen and antibody typing with additional advances in technology has brought blood transfusion to be part and parcel of clinical picture [1].

According to WHO 2016 Global Status Report on Blood Safety and Availability, Whole blood donation rate per 1000 population per year was 32.1 donations in high income countries, while it was 4.6 in low income countries. More than half of donated blood was collected in high-income countries, home to 19% of the world’s population [3].

In high-income countries, transfusion is most commonly used for supportive care in cardiovascular and transplant surgery, massive trauma, and therapy for solid and haematological malignancies. In a prospective observational study in England, Wells et al. described that 5047 units (51.6% of total during the study period) of collected blood were given to medical patients, while 40.7 and 6.3% units were given to surgical and Obstetrics and Gynecology patients respectively. On the other hand, in low and middle-income countries, it is more often used to treat pregnancy related complications and severe childhood anaemia [3–5].

Requesting blood products preoperatively for perioperative resuscitation is a common practice in surgical patients [6]. The ordering physicians usually request more units of blood than the patient will be receiving to provide a safety margin in an event of unexpected haemorrhage. In addition, preoperative ordering patterns may be more often guided by habit than clinical needs [7]. This can result in loss of blood shelf life, waste of material and human resources in blood banks, and wastage of the scarce resource i.e.- blood [8, 9]. A report from Nigeria showed 69.7% non-utilisation of cross matched blood [10].

Every year about 200, 000 surgical procedures are done in Ethiopia [11]. Tikur Anbessa Specialized Hospital (TASH) is a tertiary teaching hospital in Addis Ababa, Ethiopia. It has 550-beds and provides surgical services in the field of general Surgery, Ear Nose and Throat (ENT), Gynaecology& Obstetrics, neurosurgery and Orthopaedic Surgery.

The current practice of blood product requesting system in our set up is to perform blood typing and complete blood count for all surgical patients initially, followed by preparing a cross matched blood depending on the estimated blood need of the patient as determined by the operating surgeon, surgical resident or anaesthesiologist/anaesthetist. Cross match is requested 1 day before the operation. The blood is transported from the National blood bank and cross match is done in a day prior to the operation schedule. The blood will be unavailable for the other patients at the day of the surgery and 24 h post-surgery.

### Statement of the problem

Universal access to safe, affordable surgery when needed depends on a sufficient and safe blood supply, which is a common problem in many countries. Blood is a scarce product worldwide, and particularly in Ethiopia where donation rate is significantly disproportionate to the demand. It was reported to be one of the lowest in the world, 0.6 units per 1000 population; roughly 56, 000 units per year, as compared to 36.4 units per 1000 population in high income countries. This is despite the presence of more than 3 million births per year, which estimates 66, 000 to 230, 000 possible mothers who will require blood transfusion for postpartum haemorrhage alone [12–14]. This clearly puts the country in huge blood scarcity.

### Significance of the study

In this continual blood demand, there should be no point where the already collected blood is wasted. One of the reasons for blood wastage is preoperative excessive cross matching with inefficient utilisation. This demands us to review how efficient we are in utilising the already collected blood in our set up. A study done in Northwest Ethiopia teaching hospital has showed inefficient blood use in elective surgical patients [15].

### Literature review

Different evaluation methods have been developed to assess efficiency of blood requesting as compared to its utilisation. In 1970s Boral and Henry first used cross match to transfusion(C/T) ratio and considered appropriate blood usage if the ratio was 2.5:1 and below, while ratio of 1.0 (*all crossmatched blood is transfused*) would be ideal [16]. Using C/T ratio as a parameter, studies across the world showed inappropriate blood usage (C/T ratio > 2.5) in many countries like Malaysia, Egypt, Tanzania and Zambia with C/T ratios 5.0, 3.9, 3.7 and 2.8 respectively, while relatively better usage was reported from Ethiopia and Nepal with C/T ratio of 2.3 and 2.5 respectively [9, 15, 17–19]. Although useful for appraising the tendency to over order, this ratio neither defines the probability that a transfusion will be required for a particular procedure, nor resolves whether the number of units ordered for a procedure is appropriate [20].

In 1980 Mead et al. came up with probability of transfusion (TP) for a certain procedure which was calculated as number of patients transfused/number of patients cross matched × 100. Thirty% and above was suggested to indicate appropriate blood requesting and usage [20]. An overall TP of 20%, 11.1–25, 36.9, and 47% were reported from teaching hospitals in Zambia, Nepal, Egypt and Ethiopia respectively [15, 18, 19, 21].

Another method is Transfusion Index (TI) which indicated average number of units used per patients cross matched. A value of 0.5 or more indicates efficient blood use. It signifies the appropriateness of number of units crossmatched [22]. Similar to other indices, overall TI reports also vary across different studies, 0.4, 0.69, 0.77 from Zambia, Egypt and Ethiopia respectively [15, 18, 19].

Maximal surgical blood order schedule (MSBOS) was initiated in 1973 by Friedman and associates to increase efficiency of blood usage. It establishes a guideline on common elective procedures whether to do blood type and screen only, or do Crossmatch with recommended number of blood units [22]. It can be calculated with a simple formula: multiplying Transfusion index by 1.5 [15].

Utilisation of blood for surgical patients in terms of percentage of transfusion to cross match are very much different across different set ups. It was reported to be 18% in teaching hospital in Nepal, 35.2 in Zambia and 43.6% in Ethiopia [15, 19, 21].

Disparities in efficiency of blood utilisation in emergency and elective procedures were reflected in different studies. In studies in Ethiopia, Egypt and Nigeria, cross match to transfusion ratios were better in emergency than elective surgeries [15, 18, 23]. Belayneh et al. did a cross sectional study in Gondar University teaching hospital, in Northwest Ethiopia in 2013 and reported that overall ratios of C/T, %T, and TI index were 2.3, 47%, and 0.77, respectively. Excessive cross matching with minimal transfusion was reported in elective surgical patients and development of maximal surgical blood order schedule was recommended, while blood utilisation practices were in recommended international standards for emergency patients [15].

## Methods

### Study area

This study was done at Tikur Anbessa Specialised Hospital (TASH) where Surgical, Gynecology and Obstetrics, Neurosurgical, Orthopaedics and ENT departments’ service is being provided. The hospital is located in Addis Ababa, a capital city of Ethiopia. The city has twelve governmental and nine nongovernmental hospitals. TASH has 600 beds and offers diagnostic and treatment for approximately 370,000 to 400,000 patients per year. The hospital provides surgical procedures for about 12,000 patients per year among which about 8000 are major procedures and the rest are minor procedures. Crossmatch was requested for 747 patients over a period of 3 months. Among these 341 patients were not operated on scheduled day [surgery was cancelled].

### Study design

Cross-sectional descriptive study was done, and data was collected from blood bank charts and patient charts of TASH from December 1, 2017 to February 282,018. Training was given to data collector and supervisor by principal investigator. Data was collected from blood bank registries and patient charts of TASH. Those patients for whom blood cross matching is requested are registered in blood bank registries. A structured questionnaire will be used to extract data for each patient for whom blood is requested for perioperative resuscitation. The identification (Medical record number), age, sex, blood group of the patients, number and type of blood product requested as well as number of units taken from the blood bank was collected from these registries. By tracing the patient through his/her medical record number and the ward he/she is admitted to, remaining data was collected from patients’ charts.

### Sample size

A 3 months prospective data was collected from surgical patients for whom blood was requested between December 2017 and end of February 2018. Total number of patients who fulfilled the inclusion criteria was 406.

### Inclusion and exclusion criteria

#### Inclusion criteria

- Any patient in the specified period of time for whom whole blood/Packed RBC request will be made for the purpose of perioperative resuscitation.

#### Exclusion criteria

- Surgical patients for whom blood request was done for non-surgical use like anaemia of different cause, transfusion for medical illness.
- Scheduled patients who were cancelled after Crossmatch was done.

### Operational definitions

The following operational definitions were used for this study [16, 24].

- *Crossmatch to transfusion ratio (C/T ratio)* = number of units cross matched/number of units transfused. A ratio of 2.5 and below is considered indicative of significant/efficient blood usage.
- *Transfusion probability (TP)* = number of patients transfused/number of patients cross matched × 100. A value of 30% and above will be considered indicative of significant blood usage.
- *Transfusion index (TI)* = number of units transfused/number of patients cross matched. A value of 0.5 or more will be considered indicative of significant blood utilization.

### Data management

A structured questionnaire was used to collect data for each patient whose blood is requested for perioperative resuscitation. Patients were followed for a certain period of time until a number of units of blood transfused (Additional file 1).

### Quality assurance

Trained final year medical students and nurses were involved for data collection. The data collectors were well oriented about the objective of the study and data collection tool, procedures and approaches. Pre-test was done prior to the actual data collection among patients who were not included in the study and modifications were done accordingly. The collected data were checked for consistency and completeness regularly by the principal investigator.

### Data analysis and data quality management

The collected data were entered in to SPSS version 22.0 software. Data cleaning was done before the actual data analysis. Relevant descriptive statistics such as frequencies, percentages and ratios were carried out. Finally, the results of the study were presented using narration statements and tables.

## Results

### Profile and admission of patients

A total of 406 patients who fulfilled the inclusion criteria were included in this study. More than half, 210 (51.7%) of the study participants were females. Eighty-nine (21.9%), 65 (16.0%) and 61 (15%) of the patients were admitted and followed by Orthopedic Surgery, Urology/Endourology and General Surgery departments/units. One hundred seventy-nine (44.1%) of the patients had O^+^ blood type. The details about profile and admission of patients are presented in Table 1.

**Table 1:** Profile of patients who undergone for surgical procedures in Tikur Anbessa Specialized Hospital, December–February 2017/18 (*n* = 406)

Variables	Frequency	Percent (%)
Sex		
Male	196	48.3
Female	210	51.7
Department/Unit responsible		
Department of surgery		
General and vascular	61	15.0
Cardiothoracic	35	8.6
Neurosurgery	41	10.1
Urology/Endourology	65	16.0
Pediatric surgery	34	8.4
Orthopedic surgery	89	21.9
OBGYN		
Gynecology	49	12.1
Obstetrics	31	7.6
ENT	1	0.2
Blood type of patient		
A^+^	105	25.9
A^−^	8	2.0
B^+^	68	16.7
B^−^	68	1.0
O^+^	179	44.1
O^−^	12	3.0
AB^+^	29	7.1
AB^−^	0	0.0

### Perioperative blood work for surgical procedures

Among all study patients, 12–16 mg/dl, 10–12 mg/dl and ≥ 16 mg/dl Hg were cross matched for 251, 72 and 39 patients respectively. In addition, 12–16 mg/dl, 10-12 mg/dl and 7–10 mg/dl Hg were transfused to 25, 15 and 12 patients respectively. Cross match to transfusion ratio (C/T ratio) for all Hg categories was greater than 2.5 except one category (< 7 mg/dl). Cross match to transfusion ratio (C/T ratio) for all education level of operating surgeons was greater than 2.5, whereas cross match to transfusion ratio (C/T ratio) for estimated blood loss of 750–1500 ml and 1500–2000 ml was less than 2.5; 1.53 and 1.18 respectively. The details are presented in Table 2.

**Table 2:** Perioperative blood work versus C/T Ratio, TP and TI for patients for whom blood was cross matched for surgical procedures in Tikur Anbessa Specialized Hospital, December–February 2017/18(*n* = 406)

Variables	Cross matched patients (n)	Transfused patients (n)	Cross matched units	Transfused units	C/T ratio	TP	TI
Hg in mg/dl							
≥ 16	39	6	97	13	7.46	15.38	0.33
12–16	251	25	553	54	10.24	9.96	0.22
10–12	72	15	167	24	6.96	20.83	0.33
7–10	35	12	67	21	3.19	34.29	0.60
< 7	4	4	7	6	1.17	100.0	1.5
Education level of operating surgeon							
Consultant	306	52	698	101	6.91	16.99	0.33
Fellow	22	4	46	6	7.67	18.18	0.27
Senior resident	60	4	120	7	17.14	6.67	0.12
Junior resident	17	2	34	4	8.50	11.76	0.24
Estimated blood loss (in ml)							
< 750	372	32	810	58	13.97	8.60	0.16
750–1500	24	21	66	43	1.53	87.50	1.79
1500–2000	8	8	20	17	1.18	1.00	2.13
2000–3000	1	1	2	0	… .	1.00	0.00

### Blood utilization for surgical procedures

Among the study participants, 89, 65 and 60 patients’ blood was cross matched for Orthopedics surgery, Urology/Endourology, and General and Vascular surgery respectively. High number of cross matched unites was in Orthopedics surgery (205 units) followed by General and Vascular surgery (151 units) and Urology/Endourology (137 units). The number of transfused patients and transfused units among patients who undergone elective surgery was vary among departments/units. Seventeen, 11, and 10 transfused patients were from Orthopedic Surgery, Urology/Endourology and Neurosurgery departments/units respectively. In addition, 33, 23, and 21 units of blood were transfused in Orthopedic Surgery, Neurosurgery and Urology/Endourology departments/units respectively. Cross match to transfusion ratio (C/T ratio) was greater than two in all departments and transfusion probability (TP) was less than 30% in all departments. In addition, Transfusion index (TI) was less than 0.5 in all departments except Neurosurgery department. These findings indicate insignificant blood utilization. The details are presented in Table 3.

**Table 3:** Blood cross matching and utilisation profile for elective patients for whom blood was cross matched for surgical procedures in Tikur Anbessa Specialized Hospital, December–February 2017/18 (*n* = 406)

Variable	Cross matched patients (n)	Transfused patients (n)	Cross matched units	Transfused units	C/T ratio	TP	TI
Department of surgery							
General and vascular	60	6	151	12	12.58	10.00	0.20
Cardiothoracic	35	5	87	10	8.70	14.29	0.29
Neurosurgery	41	10	112	23	4.87	24.39	0.56
Urology/Endourology	65	11	137	21	6.52	16.92	0.32
Pediatric surgery	34	3	42	4	10.5	8.82	0.12
Orthopedic surgery	89	17	205	33	6.21	19.10	0.37
OBGYN							
Gynecology	49	8	100	13	7.69	16.33	0.27
Obstetrics	31	2	62	2	31.0	6.45	0.06
ENT	1	0	2	0	....	0.00	0.00

Key: *OBGYN* Obstetrics and Gynaecology, *ENT* Ear Nose and Throat

Overall cross match to transfusion ratio (C/T ratio) was greater than 2.5; transfusion probability (TP) was less than 30% and transfusion index (TI) was less than 0.5. These findings indicated that the overall blood utilization among patient’s undergone surgical procedures in the hospital was insignificant. The details are depicted in Table 4.

**Table 4:** Overall blood utilization among patients undergone surgical procedures in Tikur Anbessa Specialized Hospital, December-Februaury2017/18 (*n* = 406)

Blood transfusion indicators	Value	Utilization status
Cross match to transfusion ratio (C/T ratio)	898/118 = 7.6	Insignificant blood utilization
Transfusion probability (TP)	62/405 × 100 = 15.3%	Insignificant blood utilization
Transfusion index (TI)	118/405 = 0.29	Insignificant blood utilization

### Blood transfusion status and outcome of patients

Among the total patients, blood was transfused to 62 (15.3%) of the patients. Twenty one 21(5.2%) and 17 (4.2%) of patients were transfused in intraoperative and preoperative time respectively. Four hundred five (99.8%) of the patients preoperative hemodynamic status was stable and 373 (91.9%) of the patients estimated blood loss was less than 750 ml. General anesthesia was used for 277 (68.2%) of the surgeries and 307 (75.6%) of surgeries were done by consultant surgeons. Among all patients who undergone surgery, 379 (93.3%) of them were transferred to ward. The details are depicted in Table 5.

**Table 5:** Blood transfusion status and outcome of patients for surgical procedures in Tikur Anbessa Specialized Hospital, December–February 2017/18 (*n* = 406)

Variables	Frequency	Percent (%)
Transfusion status		
Yes	62	15.3
No	344	84.7
Time of transfusion		
Preoperative	17	4.2
Intraoperative	21	5.2
Postoperative	9	2.2
Preoperative and Postoperative	1	0.2
Intraoperative and Postoperative	6	1.5
No transfusion	352	86.7
Preoperative hemodynamic status		
Stable	405	99.8
Unstable with compensated shock	1	0.2
Estimated blood loss (in ml)		
< 750	373	91.9
750–1500	24	5.9
1500–2000	8	2.0
2000–3000	1	0.2
Type of anesthesia		
General anesthesia	277	68.2
Local anesthesia	129	31.8
Education level of operating surgeon		
Consultant	307	75.6
Fellow	22	5.4
Senior resident	60	14.8
Junior resident	17	4.2
Outcome of patient		
Transferred to ICU	27	6.7
Transferred to ward	379	93.3

## Discussion

Requesting blood preoperatively for perioperative resuscitation of surgical patients is a common practice. Despite its importance, overestimation of need for blood has resulted in underutilization of crossmatched blood [10]. Ethiopia is one of the countries with lowest donation rate while faced with huge demand [3]. This made it essential to use the already collected blood effectively.

Since over ordering of blood preoperatively was reported by Friedman et al. in 1970s, many studies have reported inefficient blood utilization worldwide [7, 15, 18, 23, 24]. Generally, among crossmatched surgical patients only 5–40% receives the transfusion. In our study, among 406 crossmatched patients only 62 (15.3%) were transfused indication non utilization in 84.7% of patients. This result is similar to studies from Egypt (74.8%) and India (83.9%) but far less than from Gondar, Ethiopia (56.4%) [15, 18, 24].

Different indices for evaluation of efficiency of blood utilization have been developed since the 1970s. In 1975 Boral and Henry first used cross match to transfusion(C/T) ratio and considered appropriate blood usage if the ratio was 2.5:1 and below, while ratio of 1.0 (*all crossmatched blood is transfused*) would be ideal [16]. Our study showed overall C/T ratio of 7.6 which shows insignificant blood usage. Studies across the world showed inappropriate blood usage (C/T ratio > 2.5) in many countries like Malaysia, Egypt, Tanzania and Zambia with C/T ratios 5.0, 3.9, 3.7 and 2.8 respectively, while relatively better usage was reported from Ethiopia and Nepal with C/T ratio of 2.3 and 2.5 respectively [9, 15, 17–19]. Our finding is close to but worse than many developing countries. The results of C/T ratio varied in different departments and units which showed highest C/T ratio (31.0) in obstetrics unit and lowest (4.87) in neurosurgery unit. The differences in ratios across different departments/units are because of a trend of over ordering in respective departments/units and absence of clear maximal blood ordering schedule guideline.

In 1980s Mead et al. suggested transfusion probability as additional index to evaluate efficiency of blood transfusion. Probability of transfusion (TP) for a certain procedure was calculated as number of patients transfused/number of patients cross matched × 100. Thirty% and above was suggested to indicate appropriate blood requesting and usage [20]. Overall TP from our study was 15.3%, ranging from 24.4% in neurosurgery unit and 6.5% in obstetrics unit. Our finding significantly lower compared to TP of 47% reported form Gondar, another teaching hospital in Ethiopia but relatively similar with other developing countries who reported TP of 20%, 11.1–25, 36.9%, from Zambia, Nepal, Egypt respectively [15, 18, 19, 21].

A third criteria for evaluation of efficiency of blood use is Transfusion index signifies the appropriateness of number of units crossmatched [22]. It indicates average number of units used per patients cross matched. A value of 0.5 or more indicates efficient blood use. Overall TI from our study was 0.29 which shows insignificant blood usage which is close to a study from Zambia (0.4) but far less than reports from Egypt and Northern Ethiopia (0.69 and 0.77 respectively) [15, 18, 19]. Only neurosurgical unit showed significant blood usage (TI = 0.56) and TI was lowest in Obstetrics unit (0.06).

## Conclusion

In conclusion, blood utilization practice for elective surgical procedures is inefficient. Therefore, we recommend for our hospital to develop its own Maximal Blood Ordering Schedule to improve efficiency of blood ordering and utilization.

## Supplementary information

**Additional file 1.** Efficiency of Blood Utilisation in Surgical Patients: A Prospective Study in a Tertiary Hospital.

## Data Availability

All the data generated and used in the study are available and accessible from corresponding author upon request.

## Abbreviations

C/T ratio: Crossmatch to transfusion ratio
MSBOS: Maximal surgical blood ordering schedule
TASH: Tikur Anbessa Specialized Hospital
TI: Transfusion index
TP: Transfusion probability
