# Supplementary material for: Efficiency of blood utilization in elective surgical patients
Source: BMC Health Serv Res. 2019 Nov 6;19:804. doi: 10.1186/s12913-019-4584-1 (PMC6836486; doi:10.1186/s12913-019-4584-1)
Supplement: Supplementary file 1 — Additional file 1. Efficiency of Blood Utilisation in Surgical Patients: A Prospective Study in a Tertiary Hospital. [file 12913_2019_4584_MOESM1_ESM.docx]

Research Title: *Efficiency of Blood Utilisation in Surgical Patients: A Prospective Study in a Tertiary Hospital.*

***Data collection questionnaire***

*Name of data collector _____________________ Phone: ___________________ Code:*

1. MRN: _______________
2. Sex: a. Male b. Female
3. Date of blood request (*Ethiopian Calendar*) __________
4. Date of transfusion *(Ethiopian Calendar*) ____________
5. Blood type
   - 1. A+
     2. A-
     3. B+
     4. B-
     5. O+
     6. O-
     7. AB+
     8. AB-
6. Mode of surgical admission
   1. Elective
   2. Emergency
7. Department/unit responsible for the surgical procedure (requested for cross-match)
   1. Surgery department, if yes specify the unit.
      1. General and Vascular surgery
      2. Cardiothoracic surgery
      3. Neurosurgery
      4. Urology/Endourology
      5. Paediatric surgery
   2. Orthopaedic surgery
   3. Gynecology and obstetrics
      1. Genecology
      2. Obstetrics
   4. ENT
8. Primary diagnosis: ________________________________________________________
9. Procedure to be done, specify ____________________________________________________
10. Cross match requesting and utilisation

| Type of blood | Units requested/cross matched | Number of units transfused | | remark |
| --- | --- | --- | --- | --- |
|  |  | Taken from blood bank | Transfused |  |
| A+ |  |  |  |  |
| A- |  |  |  |  |
| B+ |  |  |  |  |
| B- |  |  |  |  |
| O+ |  |  |  |  |
| O- |  |  |  |  |
| AB+ |  |  |  |  |
| AB- |  |  |  |  |

1. Preoperative blood work (in seven days before day of operation, if multiple use the latest)
   1. Haemoglobin in mg/dl: ___________
   2. Haematocrit in %: ______________
2. Timing of transfusion
   1. Preoperative
   2. Intraoperative
   3. Postoperative
3. Preoperative hemodynamic status of the patient
   1. Stable
   2. Unstable with compensated shock
   3. Unstable with decompensated shock
4. Estimated amount of blood loss during surgery
   1. <7500ml
   2. 750 – 1500ml
   3. 1500 – 2000ml
   4. 2000 – 3000ml
   5. >3000ml
5. Outcome of patient
   1. Transferred to ICU
   2. Transferred to ward
   3. Dead
6. Level of education of operating surgeon
   1. Consultant
   2. Fellow
   3. Senior resident
   4. Junior resident
7. Type of anaesthesia
   1. General anaesthesia
   2. Local anaesthesia
